# Stunt performers’ reluctance to self-report head trauma: a qualitative study

**DOI:** 10.1186/s12995-024-00401-0

**Published:** 2024-01-31

**Authors:** Jeffrey A. Russell, Elizabeth A. Beverly, Lori J. Stewart, Leslie P. McMichael, Ariana B. Senn

**Affiliations:** 1 Laboratory for Science and Health in Artistic Performance, Division of Athletic Training, College of Health Sciences and Professions, Ohio University, Athens, Ohio USA; 2 Ohio University, Heritage College of Osteopathic Medicine, Athens, Ohio USA; 3 Union of British Columbia Performers/ACTRA, Vancouver, British Columbia Canada; 4 White Wing Enterprises, Ltd., Cochrane, Alberta Canada; 5 Present Address: Feld Entertainment, Inc. , Palmetto, Florida USA; 6 School of Applied Health Sciences and Wellness, Ohio University, Grover Center E182, 45701 Athens, Ohio USA

**Keywords:** Head trauma, Head injuries, Occupational, Stunt performers, Concussion, Traumatic brain injury, Brain injuries, mTBI, Commotio cerebri

## Abstract

**Background:**

Mild traumatic brain injuries receive voluminous attention in the research literature, but this is confined almost entirely to sports and military contexts. As an occupation, performing stunts in film, television, and entertainment places the head at high risk of repetitive impact and whiplash, but stunt performers do not enjoy the same level of healthcare supervision and access as that provided to sports participants. Therefore, the aim of this study was to evaluate stunt performers’ qualitative perceptions of reporting and management of head trauma in their industry.

**Methods:**

After giving their informed consent, 87 motion picture and television stunt performers responded to a query about their views of ways to improve how stunt performers’ occupational head trauma—specifically head impacts and head whips that could cause a concussion—are reported and managed. We analyzed their responses via content and thematic analyses. Two researchers independently marked and categorized key words, phrases, and texts to identify codes that described participants’ comments. They then revised, discussed, and resolved coding discrepancies through consensus to establish inter-coder reliability. Next, we identified thematic patterns that described participants’ understanding of the stunt performer industry and what must change to facilitate reporting of head trauma. We derived themes from data that occurred multiple times, both within and across short answer responses.

**Results:**

We identified three primary themes cited by the stunt performers as needs in their industry: (1) Need to Reduce the Stigma of Reporting a Stunt-Related Injury, (2) Need to Eliminate the “Cowboy Culture,” and (3) Need to Improve the Quality of the Work Environment.

**Conclusions:**

Stunt performers are crucial members of a global entertainment industry valued at approximately US$100 billion annually. A large segment of the world’s population consumes their work in motion pictures, television, and live entertainment. When they are given an anonymous opportunity to speak, stunt performers offer insight into and recommendations for industry changes—primarily cultural and educational in nature—that could improve their physical and mental health, career longevity, and employability when they are confronted with head trauma.

## Introduction

Concussions have received unparalleled attention in sports and the military, with the former being far and away the most noteworthy to the general public because of high sports participation rates among youths and young adults, as well as the outsized visibility of university and professional sports. On the other hand, despite limited published research on head trauma in performing artists compared to sport-related head trauma [1–5], certain types of artists are at high risk for head impacts [6]. One group of performing artists intuitively with a high potential for concussions are stunt performers who engage in collisions, falls, fights, explosions, vehicular crashes, and similar high energy, high velocity activities in film, television, and entertainment theme parks. Their role is to assume the risk of a stunt, and often from a headline actor; that is, they must make a stunt look realistic with as low a chance of injury as possible.

Moreover, these performers are essential to creative success in an entertainment industry that knows few bounds in terms of availability to most of the world’s population. It is likely that many readers of this article are well-acquainted with the entertainment media in which these industrial athletes perform. Clearly the stakes are high as it is a global business valued at approximately US$100 billion. Curiously, however, head injuries in stunt performers have been reported heretofore by celebrity entertainment outlets [7–12] rather than in scientific literature.

Unfortunately, stunt performers do not routinely have on set access to specialized healthcare for their head trauma that clearly require a high standard of care. While they do have first aid provided for standard injuries and emergency care when necessary, the unique care required for head injuries is generally not available on set. This is diametrically opposite the situation in professional sports. Stunt performers have reported 80 to 100% prevalence of head impacts during their careers [5, 13, 14], but offer a number of reasons why they do not report those injuries, including not realizing the seriousness of the injury and its long-term consequences, a “tough it out” culture; pressure from supervisors, directors, and producers; and a variety of fears: of missing work, downgraded reputation, and losing future work opportunities [5]. In addition, the simple absence of healthcare professionals qualified to manage the evaluation and treatment of stunt performers’ concussions is a concern.

Prior research suggests that most stunt performers recognize and accept that head injuries are part of their occupational hazards; however, objectively they show diminished mental health scores, while their physical health scores remain high [5]. In conceptualizing the present study, we believed that when given an anonymous opportunity to express their feelings, stunt performers’ comments would reflect sensible beliefs about head trauma, as well as concerns about how head trauma is managed in their industry.

In the absence of qualitative concussion research with performing artists, the sports concussion qualitative literature highlights participants’ important beliefs and feelings about concussions in a sports context, as well as their approaches to managing concussions. In studying retired professional rugby players, Daly et al. [15] suggested that their participants did not fully understand concussions nor were they inclined toward proper management, largely because of cultural norms within rugby that regard concussions as part of the game. The researchers found that this led to trivialization of concussion occurrence—including among coaches—that negatively affected attitudes and treatment seeking behaviors.

Weber Rawlins et al.’s [16] qualitative study of university athletes reported the importance of prior experience with concussions. They found that potential detriments to the athletes’ health played a role in the formation of their attitudes about concussion, as did the involvement of various people in their support systems, including coaches, parents, and athletic trainers. Notably in this sports setting, the availability of athletic trainers—healthcare professionals who are skilled in evaluating and managing concussions, who work closely with sports teams, and who typically have excellent relationships with the athletes under their care—was suggested as important to improving the reporting of concussions.

In light of prior research and remaining gaps in our knowledge (specifically about head trauma in stunt performers), the purpose of this study was to evaluate stunt performers’ qualitative perceptions of reporting and management of head trauma in their industry. We sought to better understand their reporting hesitancy, their beliefs about concussions within their industry, and their mental processes for dealing with both the risk and occurrence of concussions.

## Method

As part of a larger, cross-sectional survey study examining stunt performers’ head impact history, their willingness to report head injuries, and any reasons they might have for not reporting those injuries, this qualitative analysis explored considerations that would help stunt performers’ feel more comfortable reporting their head impacts/head whips to stunt coordinators or healthcare professionals. The Ohio University Institutional Review Board approved the protocol and all recruitment procedures and materials for this study (approval number 20-X-148); the research was conducted according to the Declaration of Helsinki. All participants provided informed consent prior to participation. Especially notable about this project is our incorporation of community-engaged co-investigators from the stunt performance community—who also are co-authors (LJS, LPM)—as a means to ensure appropriate representation of the interests of that community [17].

### Participants

Eighty-seven participants from the 216 current or former stunt performer members of the Alliance of Canadian Cinema, Television and Radio Artists (ACTRA) who undertook a survey research project reported previously [5] formed the participant group for this study. These participants self-selected into the study based on their willingness to offer the qualitative comments we requested.

ACTRA is a union that represents these actors. Exclusion criteria included individuals who were not members of ACTRA, not stunt performers, under the age of 18 years, or could not read and write in English. The study opened in February 2021 and closed in April 2021. Participation in the study was completely voluntary and anonymous.

### Data collection

All 87 participants responded to the following open-ended question: “Please provide any comments you think would help stunt performers feel more comfortable about reporting their head impacts/head whips to stunt coordinators or healthcare professionals.” In addition, we collected demographic data, including age, gender, years as a stunt performer, history of a head injury, and number of head injuries, as well as the mental component score of the Optum SF-12 health-related quality of life survey (QualityMetric, Johnston, Rhode Island, USA). To maintain anonymity, no identifying information (e.g., name, email address) was collected. For the purposes of this study, “head trauma” was defined as any impact to the head or whiplash that impelled a force to the head, as both of these are mechanisms of concussion [18]. Furthermore, this is why we employed the terminology “head impacts/head whips.”

Participants completed the survey online via the electronic survey service Qualtrics (Qualtrics, Inc., Provo, Utah, USA). Qualtrics permitted the research team to download participants’ survey responses into a spreadsheet without including identifying information. The informed consent was given via the online survey prior to proceeding. No researchers were present when potential participants decided to participate to ameliorate any perceived pressure to do so. Participants with questions about the study were directed to email or phone the principal investigators. Participation in the entire study lasted approximately 15–20 min. Participants received no compensation for completing the survey.

### Data analysis

All demographic and mental component score data were analyzed using descriptive statistics. We analyzed the open-ended short answer question via content and thematic analyses [19, 20]. First, two researchers (JAR, EAB) independently marked and categorized key words, phrases, and texts to identify codes that described participants’ comments that would help stunt performers feel more comfortable about reporting their head impacts/head whips to stunt coordinators or healthcare professionals. The researchers revised, discussed, and resolved coding discrepancies through consensus to establish inter-coder reliability. The Cohen’s kappa coefficient for the interrater agreement between the two coders was 0.894, indicating excellent agreement. Second, we conducted thematic analysis to identify patterns across the data [21]. The selected themes described participants’ understanding of the stunt performer industry and what must change to facilitate reporting of head impacts/head whips. We derived themes from data that occurred multiple times, both within and across short answer responses.

## Results

### Descriptive statistics for demographics

A total of 87 out of the 216 participants (response rate = 40.3%) responded to the open-ended question. There were 59 males and 28 females. Their mean age was 44.7 ± 10.7 years and their years of experience in the stunt industry was 17.2 ± 9.7 years. All 87 participants reported they had received at least one stunt-related head impact during their career. They did not exhibit statistically significant differences in age or stunt experience compared to the individuals who did not participate in this qualitative study. Their mean score on the 0–100-point SF-12 mental component score was 47.8 ± 9.2 (range = 23.6–62.9; CoV = 19%). The mental component score median was 49.5. Of the 87 participants, 31 (36%) scored below 45.0 on the mental component score, and 21 (24%) scored below 40.0.

### Recurrent themes

The participants’ answers to the question posed were categorized into three main themes that express key areas where the stunt performers perceive improvement strategies are needed to make their occupational context more manageable from a health and safety perspective. These themes were the need to reduce the stigma of reporting a stunt-related injury, the need to eliminate the “cowboy culture,” and the need to improve the quality of the work environment. Results for each are provided below.

#### Need to reduce the stigma of reporting a stunt-related injury

Comments by our respondents suggest stigma associated with reporting work-related injuries is widespread:

Remove the ‘tough it out’ stigma. Recognize that, while performance athletes, performers are not super-human. We all bleed the same blood. More overall care in the industry [is needed].

I was clearly told that if I hit my head I’m fired. And this was true.

The biggest issue is fear of being removed from stunt rosters. It is an extremely competitive industry already. If the difference between working or not working is a minor injury, most performers including myself will choose to work.

The problem with reporting a head injury is that when this happens and a performer reports it, this spreads through the community and production and the performer fears that they will not work due to this.

There is a fear of not being hired if an injury occurs. I have no idea how to make performers feel more comfortable.

Keeping the discussion going helps to take the stigma away from performers feeling afraid to report injuries. Things are changing—it is a bit of an older school attitude to hide injuries. Also, the thing that holds performers back is feeling that you are letting the [stunt] coordinator down—that it will look bad for them and you say nothing out of loyalty to them. So perhaps if [stunt] coordinators are instructed to continue to let performers know that there is no shame in admitting injury.

It is clear from these comments—representing many others that were similar—that stunt performers often carry a deep concern that an injury reporting-related stigma will negatively affect their ability to continue their professional livelihood.

Occasionally participants sought to overcome stigma by stating their desired solutions, as in these examples:

All head impact/head whips resulting in any adverse symptoms should be reported to production for their own protection should future issues arise.

Reminder from stunt coordinator to stunt performer, when there is potential for head injury or whip during a scene, that stunt performer not let ego get in way of reporting to coordinator that their bell was rung. Immediately after being asked after a hard take if they are okay to go again, performer should never feel the need to hide injury just to please production or to act tough.

Elaborate in safety meetings about opening up to mental health issues without fear of ridicule. Allowing this to happen would provide a more welcoming work environment to all parties.

#### Need to eliminate the “Cowboy culture”

“Cowboy culture,” derived primarily from ideas reminiscent of the *vaqueros* of Mexico and the ensuing cowboys of the western United States, is understood to be an environment where, among other characteristics, displaying emotional and physical toughness is a high priority. Our respondents related experiences consistent with feelings of needing to survive in a culture where the ability to hide pain, resist adverse circumstances, and not report injuries were valued. Many also seemed resigned to the fact that the culture has little chance of changing for the better. Comments by our participants related to this included:

I think it starts with stricter standards for stunt coordinators. The stunt industry is dangerous because it is still an old boys network that is all about keeping the money in the family and hiring your friends rather than opening up opportunities for the best qualified experts to manage stunts—especially when it comes to issues that particularly affect women—such as inadequate protective wardrobe.

It is highly unlikely that any performer is ever going to volunteer injury information that could (and will) make its way through the community and lose them work.

All I’d say is I think it’s important the medics [non-physician, on set healthcare providers] have the ‘balls’ (don’t know how else to word it) to step in and say, ‘No, this person might be hurt; we should stop this.’ I know a doctor would do that. I’ve witnessed medics go, ‘Oh, I don’t know, it might be okay,’ when it really, absolutely should have been stopped. The performer will almost always keep going and sometimes they don’t even know they’re hurt. The medics need to be able to step in similar to the doctors at MMA [mixed martial arts] fights and what not.

#### Need to improve the quality of the work environment

Our stunt performer participants readily recognized facets of their workplaces that are less than ideal with respect to head injury care. Some of the responses presented previously may overlap with work environment-related concerns. There are several ways a work environment can be improved, but any improvement should accrue a net benefit to the workers. This is particularly important where health and safety are concerned, as one respondent indicated:

Worksafe [occupational health and safety] rep should be mandatory on set for ALL wirework to make sure rehearsals/takes are stopped after 2 bad head-whips; use catch pads to prevent this. Proper ceiling heights for angle of pull and/or high lines—plenty of pads if landing not visible. Rep would speak up on behalf of performer and [stunt] coordinator as they may both be concerned for their careers. [Unsafe riggers] could be observed as incompetent and dangerous and stopped from rigging.

Other noteworthy and representative comments we received pertaining to challenges in work environment were:

Somehow with the inexperience of the [city name withheld for confidentiality] stunt coordination and the inappropriate hiring of inexperienced stunt performers, with personal reason not to hire the experienced, proven-on-film performers, is a scary and disturbing trend. The producer may have to vet the performers over the coordinator, or the [workers’ union] member suspended, if coordination is the cause of these irreversible and serious consequences.

Performer readiness is the best prevention and instills confidence to report injuries without fear of losing work. There is no standardized training for stunt performers and often they are hired based on convenience and politics without proper due diligence. Lack of education and training in stunt departments is the largest contributor to accidents and it necessitates cover-ups to save face.

Performers can control the possibility of head injuries for most stunts themselves. The exception is wirework—performers have little control. The ONLY way to prevent wirework head injuries is to have an educated WCB [Workers Compensation Board] representative for ALL wirework who has NEVER worked as a performer. The majority of stunt coordinators are too greedy, afraid, and narcissistic to be safe. Performers are afraid of losing work. A WCB rep saves the performer the embarrassment and anxiety of reporting or stopping.

One example of a proposed work environment improvement formulated by our stunt performer respondents was, “Athletic therapists should be on set. Paramedics are great but sports-oriented health professionals should be the norm during stunts. (Not just for head injuries but for all other issues that may not need emergency transport or pharmaceutical aid).” [An athletic therapist is a Canadian healthcare professional, the U.S. equivalent of which is an athletic trainer.] A related suggestion offered by a participant was, “There needs to be an on set health and safety liaison who fills out paperwork and follows up with stunt performers.”

## Discussion

The purpose of this study was to evaluate stunt performers’ qualitative perceptions of reporting and management of head trauma in their industry. Our analysis of anonymous comments provided by stunt performers provided key insights into their profession. Our sole motivation for conducting this research was to identify areas of the stunt industry where improvement in head safety could yield benefits to work conditions while lowering morbidity and lengthening career longevity of stunt performers in the motion picture and television field. In view of some of our results, it is pivotal that this project presents stunt performers’ perceptions of head injury risk and management in their own words, the first known research effort to give these workers a voice.

Moreover, this is an important topic to society considering in 2022 the worldwide market value of the film and entertainment industry was US$95.45 billion, with 35% of this value based in North America [22]. The global value is predicted to grow through 2030 at a compound annual growth rate of 7.2% to reach $169.68 billion [22]. Thus, the industry in which stunt performers work is enormous and global. It seems likely that at least once per week, and often many more times, most readers of this article partake of some type of entertainment where stunt performers are crucial to success.

### Demographics

As a prelude to understanding our stunt performer participants’ comments about head injuries in their profession and their overall well-being, it is insightful to examine some of their demographic data. Our respondents were accomplished stunt performers, with a mean of more than 17 years of experience. We thus expected their comments to be both reasoned and reflective of the experiences of seasoned veterans. Moreover, they all had sustained one or more stunt-related head impacts; therefore, their commentaries were not postulating about nebulous concepts—they knew the conditions of their work and the resulting effects on performancefrom personal experience.

### Need to reduce the stigma of reporting a stunt-related injury

Based on our respondents’ comments, stigma associated with reporting work-related injuries was widespread. Often stigmas are multi-factorial and related to mental health [23], though mental health stigmas in the workplace have received insufficient research attention [24]. Our participants were not asked to respond in a mental health context, though stigmas often have mental health manifestations [23].

Stunt performers often carry a deep concern that an injury reporting-related stigma will negatively affect their ability to continue their professional livelihood. With regard to concussion, failure to seek appropriate care may lead to increased morbidity and delayed recovery [25, 26]. Thus, the perceived nature of their industry is at odds with stunt performers’ need for their head injuries to be managed according to contemporary medical practices, such as those applied in sports [18].

Published occupational health and safety research on stunt performers is, to our knowledge, scant at best. One prior study [5] is the only known published investigation into concussions in the stunt industry. However, a study of a variety of occupations examined the effects of employees feeling under pressure to maintain a safe workplace secondary to fear of negative consequences for not doing so [27]. The researchers suggested that this type of pressure is associated with reduced safety compliance and suboptimal mental health.

### Need to eliminate the “Cowboy culture”

Dary [28], in his book *Cowboy Culture: A Saga of Five Centuries*, describes cowboy culture as “…a culture that was learned. It was a culture based on mobility, custom, and the survival of the fittest” (p. xi). A contemporary example of this in science was cited by Nadis [29], who reported about women scientists’ fight against a predominantly male cowboy culture that requires them to work harder than their male counterparts. In the context of our research, generally speaking, our participants related experiences consistent with feelings of needing to survive in an environment that can be labeled “Cowboy Culture.” This atmosphere is one where an expectation exists requiring stunt performers to ignore pain, injury, and other adverse circumstances (to the point of not outwardly expressing these in any way) in favor of “The Show Must Go On.” While we did not specifically address the emotional, psychological, and physical tolls of this *modus operandi* in our study, we presume there is a substantial cost in these attributes in many stunt performers.

### Need to improve the quality of the work environment

Rossol [30] distinguishes between health and safety in the film, television, and theater professions by asserting that health is more nebulous and difficult to contend with because health-related conditions are more difficult to recognize and prove. She suggests that health relates to exposure to environmental toxins and particulates, injurious sound levels, temperature extremes, and other physical characteristics that may be present at work. Safety, in her view, relates to accidents such as traumatic injuries (cuts, bruises, fractures, and the like that result from falls), working with hazardous equipment, etc.

In such a framework, concussions may straddle between health and safety by eliciting components of both. Clearly head trauma is a safety issue that accompanies how stunts are generated. No one disputes stunt performers’ tendency for accidental head contact with equipment and other actors. Based on the responses of our participants, however, approaching concussions from a health standpoint is, indeed, more challenging (as Rossol proposed). Resulting brain impairments typically are less apparent (one cannot, after all, directly observe brain tissue being injured, as can be done with, for example, a bleeding wound) and they also can be hidden by not reporting symptoms. Moreover, repetitive head impacts have been associated with other health concerns, in particular the onset of chronic traumatic encephalopathy [31] and declines in mental health [32–34].

Aligning with the history and practices of most North American professional sports teams, as well as many university and high school teams, the presence of athletic therapists, athletic trainers, or other healthcare professionals conversant with concussion diagnosis and management would likely benefit the stunt industry. One of the specific areas of clinical education for these practitioners is concussion identification and management [35–37]. As a minimum starting point, the incorporation of the Concussion Recognition Tool [38, 39], a resource designed to allow non-medical personnel to make a decision about further participation by and healthcare referral for individuals who sustain a head impact, is certainly warranted and can be implemented at virtually no cost and a minimum of training. Educational programming such as the Concussion Awareness Training Tool [40] may also be beneficial.

Finally, in reference to a suggestion by one of our participants that a health and safety liaison be present on set during stunt work, this is essentially similar to a professional patient advocate in traditional healthcare environments, an increasingly useful and important member of a medical care team [41, 42]. A professional like this, if specially trained and strategically located, could serve as an additional advocate in the context of stunt performers who are injured at work. Some productions (usually ones with large budgets) hire certified safety coordinators or safety officers to oversee all departments. These individuals are present during stunt rehearsals and while filming. They are not stunt performers, but they serve as advocates for performers and function as liaisons between performers and production to help mediate any hierarchical issues with stunt coordinators. Hiring this type of individual, regardless of production budget size, may provide substantial benefit in situations where head injuries can occur.

### Limitations

Limitations of our study include the cross-sectional design, participant self-selection, self-reported data, the potential generalizability of our findings, and the nature of the qualitative inquiry. The cross-sectional design of the study does not capture how and when stunt performers formed their views about injuries and safety in the workplace environment. Future research with a larger, more heterogeneous sample should follow stunt performers longitudinally throughout their careers to discern formative associations by year in profession, perceptions, and head trauma.

The self-reported findings may be susceptible to selection and social desirability biases. Stunt performers who volunteered to participate in the study may have been more willing or motivated to answer questions about head trauma. To minimize bias, the researchers informed participants that their responses were anonymous. Further, the researchers emphasized the voluntary nature of participation and explicitly told the participants that their responses would have no bearing on their professional standing by being linked back to their identity nor revealed to their stunt coordinators, producers, or directors.

Whereas our participants formed a subset of a sample from another study, they may not adequately represent all stunt performers. As a general principle, it is extraordinarily challenging to secure stunt performer participants because they do not like to discuss their injuries and they are suspect of how the data will be used.

Lastly, participants provided brief responses to a single short answer question. Additional research should involve the collection of in-depth qualitative data via semi-structured interviews to further understand stunt performers’ experiences with head trauma and how these experiences have influenced their career and emotional well-being.

## Conclusions

This study examined health management related to head injuries in stunt performers, a prominent and influential group of workers in light of the global popularity of the entertainment industry and the key role played in that industry by stunt performers. While head impacts have been shown to be prevalent in this population, stunt performers receive far less attention for injuries resulting from those impacts, including mild traumatic brain injuries, than their counterparts in, for example, sports. Thus, improvement in concussion management for stunt performers is important because the pervasive presence of stunts in entertainment creates a rare worldwide societal influence that must be preserved by mitigating risks and increasing on set access to high quality, specialized concussion care for these industrial-athlete workers. Notably, this study represents the first known research where stunt performers have been given a voice; they expressed in their own words how head injuries are regarded and managed in their work environment.

In conclusion, stunt performers in film, television, and other entertainment media report several concerns about their susceptibility to head trauma coupled with perceived deficiencies in the safety of their work environments and a reticence to report their head impacts. Whereas scientific inquiry of head injuries in stunt performers appears to be barely starting its infancy (despite the overtly physical, high energy, high impact performance requirements of this profession), themes from this research lay the foundation for follow-up investigations, particularly in the areas of injury reporting stigma, the prevailing existence of a “cowboy culture,” the need for workplace improvements that include specialized healthcare, and mental health, particularly as it relates to occupational head trauma. Few, if any, industries are as widely consumed on a daily, global basis as film, television, and entertainment in general—all of which depend heavily on stunt performers. This reinforces the importance of addressing the health and safety of stunt performers as key members of the entertainment industry.

## Data Availability

The datasets used and/or analysed during this study are available from the corresponding author on reasonable request.

## Abbreviations

ACTRA: Alliance of Canadian Cinema, Television and Radio Artists
MMA: Mixed martial arts
WCB: Workers Compensation Board
